# Evaluation of a Point-of-Care Feline Immunodeficiency Virus (FIV) Test Kit (RapidSTATUS™ FIV) to Determine the FIV Status of FIV-Vaccinated and FIV-Unvaccinated Pet Cats in Australia

**DOI:** 10.3390/vetsci9110618

**Published:** 2022-11-08

**Authors:** Ashley Cheang, Mark E. Westman, Jennifer Green

**Affiliations:** Sydney School of Veterinary Science, Faculty of Science, The University of Sydney, Sydney, NSW 2006, Australia

**Keywords:** antibodies, Australia, diagnosis, feline immunodeficiency virus, FIV, shelters, vaccination, veterinary science, DIVA

## Abstract

**Simple Summary:**

This study evaluated a commercial point-of-care (PoC) feline immunodeficiency virus (FIV) test kit (RapidSTATUS™ FIV) for its accuracy in determining the FIV status of FIV-vaccinated and FIV-unvaccinated pet cats in Australia. In countries where FIV vaccination is used, veterinarians need a PoC kit that will produce a negative result for a FIV-uninfected cat, even if the cat is FIV-vaccinated or the FIV vaccination history is unknown, since incorrect diagnoses can impact negatively on the welfare of cats. FIV PoC kits also need to produce positive results in FIV-infected cats to help with appropriate management and to enable strategies to be implemented to prevent other cats from becoming FIV-infected. Results presented here show RapidSTATUS™ FIV to be highly accurate (98.8−100%) in a range of FIV-vaccinated and FIV-unvaccinated scenarios. Therefore, Australian veterinarians can reliably use RapidSTATUS™ FIV to rapidly and accurately determine the FIV status of all cats.

**Abstract:**

Feline immunodeficiency virus (FIV) is a retrovirus that can cause immunosuppression, co-morbidities, and neoplasia in infected cats, and is commonly tested for in veterinary clinics and animal shelters in Australia. FIV diagnosis using point-of-care (PoC) kits to detect FIV antibodies in Australia is complicated by the commercial availability of an inactivated whole-FIV vaccine. The aim of this study was to determine the accuracy of the RapidSTATUS™ FIV antibody test kit in FIV-vaccinated and FIV-unvaccinated cats in Australia. Plasma from pet cats of known FIV vaccination and FIV infection statuses (*n* = 361), comprised of 57 FIV-uninfected cats annually vaccinated against FIV, 10 FIV-uninfected cats with lapsed FIV vaccination histories, 259 FIV-unvaccinated/FIV-uninfected cats, and 35 FIV-infected cats, was tested. RapidSTATUS™ FIV testing had sensitivity of 97.1% (34/35) and specificity of 100% (326/326), with an overall accuracy of 99.7% (360/361). Additional testing was undertaken using plasma from FIV-uninfected cats recently administered a primary FIV vaccination course (*n* = 12) or an annual booster FIV vaccination (*n* = 10). RapidSTATUS™ FIV was 98.8% (81/82) accurate and 100% (32/32) accurate in cats recently administered primary or annual FIV vaccinations, respectively. The high level of accuracy of RapidSTATUS™ FIV (98.8–100%) therefore establishes this PoC kit as a DIVA (differentiating infected from vaccinated animals) test. RapidSTATUS™ FIV is recommended to aid animal shelters, veterinarians, and researchers in Australia to accurately determine FIV infection status, irrespective of FIV vaccination history.

## 1. Introduction

In Australia, approximately 15% of adult pet cats with outdoor access are infected with feline immunodeficiency virus (FIV) [1,2,3]. The outcome for a cat that tests FIV-positive at an Australian animal shelter can vary from being adopted to being euthanized [4]. It was reported that only 35% of Australian rehoming organisations performed additional testing after obtaining a positive point-of-care (PoC) antibody test result [4], despite major retroviral guidelines recommending confirmatory FIV testing [5,6]. Sadly, 4 out of 17 (23.5%) Australian shelters surveyed reported euthanizing FIV-positive cats [4]. The accuracy of the FIV test kit used is therefore particularly crucial in shelter medicine, as it could result in a decision to euthanize misdiagnosed cats [7,8]. Accurate FIV diagnosis is also important for the welfare of client-owned cats, with FIV-infected cats recommended to be housed entirely indoors, a recommendation with the potential to negatively impact on their welfare if not appropriately managed [9,10,11,12].

Since 2004, an inactivated whole-virus vaccine to protect against FIV has been commercially available in Australia (Fel-O-Vax^®^ FIV; Boehringer Ingelheim Animal Health). The same vaccine has also been available in New Zealand since 2004 and Japan since 2008 [13]. Introduction of this vaccine posed a diagnostic challenge since the production of antibodies following FIV vaccination, and in response to FIV infection, were indistinguishable by tests available at the time (SNAP^®^ Combo FIV/FeLV, IDEXX Laboratories, Westbrook, ME, USA; and Western blot analysis) [8,14,15,16].

More recently, Witness^®^ (Zoetis Animal Health, Lyon, France) and Anigen Rapid^®^ (BioNote, Gyeonggi-do, Korea) FIV PoC antibody test kits were reported to have high levels of accuracy (98.0%; 100%), sensitivity (100%; 100%), and specificity (98%; 100%), respectively, in a large Australian cohort containing FIV-vaccinated cats [14]. Similar results were found in USA, confirming the ability of these kits to differentiate FIV-infected cats from FIV-vaccinated cats [17]. PCR testing can determine FIV status irrespective of FIV vaccination status, with one commercially available assay available in Australia (FIV RealPCR™, IDEXX Laboratories, Brisbane, Australia) having 98.3% accuracy, 92% sensitivity, and 99% specificity [14]. PCR testing, however, currently involves a 1–3 day turnaround for results and is substantially more expensive than PoC testing, making it unsuitable for rapid FIV screening (e.g., in animal shelters).

RapidSTATUS™ FIV (Biotech Laboratories, Rockville, MD, USA) is a FIV PoC kit that, according to the manufacturer, can accurately determine FIV status regardless of FIV vaccination history; however, results from independent testing are not available. As with all diagnostic tools, veterinarians need to be confident in any claims made by the manufacturer [6]. The aim of this study was to determine the accuracy of RapidSTATUS™ FIV in a mixed cohort of FIV-vaccinated and FIV-unvaccinated cats in Australia.

## 2. Materials and Methods

Residual plasma samples from three previous Australian studies stored at −80 °C were thawed and used for RapidSTATUS™ FIV testing [14,18,19]. All cats lived with their owners for the duration of the study.

### 2.1. Study Population 1—Cats with a Mixed FIV Vaccination History (n = 361)

This population comprised 57 FIV-uninfected cats annually vaccinated against FIV, 10 FIV-uninfected cats with lapsed FIV vaccination histories, 259 FIV-unvaccinated/FIV-uninfected cats, and 35 FIV-infected cats. These pet cats consisted of non-pedigreed (*n* = 314) and pedigreed breeds (*n* = 47), with ages ranging between 6 months and 18 years (median 7 years; interquartile range [IQR] 5–10 years) [14]. FIV vaccination history was determined by the interrogation of clinic medical records. Plasma samples were collected during routine visits to veterinary clinics (e.g., vaccinations) for a previous FIV vaccine field effectiveness study [20].

FIV infection status was determined using a complex algorithm that involved consideration of three PoC antibody test results (SNAP^®^ Combo, Witness^®^ and Anigen Rapid^®^) from fresh ethylenediamine tetraacetic acid (EDTA) whole blood, a real-time (q)PCR result (FIV RealPCR™), and, in rare discordant cases, virus isolation performed at the University of Florida or the University of Glasgow [14].

### 2.2. Study Population 2—Cats Administered a Primary FIV Vaccination Course (n = 12)

The primary FIV vaccination study comprised 12 pet cats (4 kittens < 6 months, 8 cats > 6 months), with ages ranging between 3 months and 7.6 years (median 1.6 years, IQR 0.5–2.5 years). The cohort consisted of 7 males and 5 females; 10 cats were non-pedigreed and two were pedigreed (both Ragdolls). All cats were neutered. Cats were determined to be FIV-unvaccinated by the interrogation of clinic medical records [18].

Cats were confirmed FIV-uninfected on day 0 using fresh EDTA whole blood and FIV PoC antibody kits from different manufacturers (Witness^®^ and Anigen Rapid^®^), and a qPCR assay (FIV RealPCR™). qPCR testing was also undertaken on day 238 to confirm cats were FIV-uninfected for the entire study period [18].

Administration of Fel-O-Vax^®^ FIV subcutaneously into the inter-scapular space, as outlined in the manufacturer’s guidelines, occurred on day 0, 28, and 56. Blood was collected for FIV testing before FIV vaccination.

The plasma samples collected on day 0, 14, 28, 42, 56, 70, 84, 98, 140, and 238 were tested with RapidSTATUS™ FIV [18]. There were 21 samples of insufficient volume, leaving 82 samples available for testing with RapidSTATUS™ FIV.

### 2.3. Study Population 3—Cats Administered an Annual Booster FIV Vaccination (n = 10)

The annual FIV vaccination study comprised 10 non-pedigreed pet cats with ages ranging between 1.3 and 10.8 years (median 6.0 years, IQR 2.8–8.7 years). The cohort consisted of 8 males and 2 females. All cats were neutered [19].

FIV vaccination history was determined by the interrogation of clinic medical records. Six cats had received their first FIV vaccination before reaching 6 months-of-age and, as per manufacturer’s guidelines, were not FIV tested prior to administration of their first FIV vaccine. The remaining four cats were given their first FIV vaccination after the age of 6 months and had FIV antibody testing performed prior to vaccination using a PoC test kit (SNAP^®^ Combo).

Primary and annual FIV vaccinations were administered according to the vaccine manufacturer’s guidelines. At the time of sampling for this study population, the median number of annual FIV vaccinations these cats had received was two (range 0–7, IQR 1–2.3), and the median time since their last annual FIV vaccination was 362 days (range 319–396 days; IQR 324–372 days) [19].

FIV-uninfected status was confirmed on day 0 using fresh EDTA whole blood and FIV PoC antibody kits from different manufacturers (Witness^®^ and Anigen Rapid^®^), and a qPCR assay (FIV RealPCR™). qPCR testing was also undertaken on day 42 to confirm cats were FIV-uninfected for the entire study period [19].

Administration of Fel-O-Vax^®^ FIV subcutaneously into the inter-scapular space, as outlined in the manufacturer’s guidelines, occurred on day 0. Blood was collected for FIV testing before FIV vaccination.

The plasma samples collected on day 0, 14, 28, and 42 were tested with RapidSTATUS™ FIV [19]. There were 8 samples of insufficient volume, leaving 32 samples available for testing with RapidSTATUS™ FIV.

### 2.4. RapidSTATUS™ FIV Testing

RapidSTATUS™ FIV is a portable lateral flow test kit made of a nitrocellulose membrane that detects antibodies directed against FIV transmembrane glycoprotein gp40, as with Witness^®^ and Anigen Rapid^®^ tests [2]. RapidSTATUS™ FIV testing was performed by two testers blinded to the FIV vaccination/infection status of each cat (A.C. and J.G.). If the result window did not show lateral flow of solution after 2 minutes, an extra drop of buffer solution was added and/or the test kit was tapped a couple of times, as per manufacturer’s instructions. There was 100% test agreement between testers.

### 2.5. Statistical Analysis

Test sensitivity was calculated using the formula (sensitivity = number of true-positives/[true-positives + false-negatives] × 100).

Test specificity was calculated using the formula (specificity = number of true-negatives/[false-positives + true-negatives] × 100).

Overall test accuracy was calculated using the formula (test accuracy = [true-positives + true-negatives]/[true-positives + true-negatives + false-positives + false-negatives] × 100) [21].

In addition, 95% confidence intervals (CI) were calculated using Microsoft Excel^®^ (Microsoft, Redmond, WA, USA).

## 3. Results

### 3.1. Study 1—Cats with a Mixed FIV Vaccination History (n = 361)

There were 326 true-negative, 34 true-positive, 1 false-negative, and 0 false-positive FIV results recorded with RapidSTATUS™ FIV testing (Table 1).

Of the FIV-infected cats (*n* = 35), the RapidSTATUS™ FIV test kit was clearly positive for 29 samples, faintly positive for 4 samples, very faintly positive for 1 sample, and negative for 1 sample. The single false-negative result recorded was in a FIV-unvaccinated cat.

No FIV-vaccinated/FIV-uninfected cats tested FIV-positive, while all three FIV-vaccinated/FIV-infected cats tested FIV-positive.

RapidSTATUS™ FIV had a sensitivity of 97.1% (34/35; 95% CI 91.6 to 100) and specificity of 100% (326/326), with an overall accuracy of 99.7% (360/361; 95% CI 99.2 to 100).

### 3.2. Study 2—Cats Administered a Primary FIV Vaccination Course (n = 12)

There were 81 true-negative results and 1 false-positive result recorded with RapidSTATUS™ FIV testing in cats recently administered a primary FIV vaccination course (i.e., 98.8% [81/82] accurate; Table 2). The single false-positive result occurred 2 weeks after the third primary FIV vaccine had been administered (Day 70) in a 1.5 year old female neutered cat, and was recorded as a very faint-positive result.

### 3.3. Study 3—Cats Administered an Annual Booster FIV Vaccination (n = 10)

There were 32 true-negative results recorded with RapidSTATUS™ FIV testing in cats recently administered an annual booster FIV vaccine (i.e., 100% [32/32] accurate; Table 2).

## 4. Discussion

In order to resolve the challenge of determining if a cat is FIV-infected, veterinarians need a PoC kit that will produce negative results for a FIV-uninfected cat, even if the cat is FIV-vaccinated or the FIV vaccination history is unknown. The latter is often the case for cats surrendered to rescue facilities or animal shelters in Australia. In the current study, RapidSTATUS™ FIV was highly accurate when determining the FIV status of cats, regardless of their FIV vaccination history, with an accuracy of 99.7% in cats with a mixed FIV vaccination history, 98.8% in cats recently receiving a primary FIV vaccination course, and 100% in cats that recently received an annual FIV vaccination booster. Therefore, Australian veterinarians can reliably use RapidSTATUS™ FIV to rapidly and accurately determine the true FIV status of all cats. Given the findings of this study, RapidSTATUS™ FIV can be considered a DIVA (differentiating infected from vaccinated animals) test for FIV, as has also been suggested for Anigen Rapid^®^ and Witness^®^ FIV PoC kits, and some other diagnostic tests for animal diseases [17,22]. Nonetheless, following any positive FIV result, confirmatory testing (e.g., with a second PoC test kit from a different manufacturer, or PCR testing) is recommended by the Australian and New Zealand FIV Guidelines [6].

A limitation of the current study was the use of samples that had undergone several freeze-thaw cycles. It is possible that antibody degradation occurred while samples were stored and transported at −80 °C. Antibodies are generally stable long term (i.e., years), particularly when stored at −80 °C, and slow thawing (i.e., on ice) is recommended to minimize antibody instability [23]. In another study, it was reported that (according to the manufacturer) hemolysis, freezing, thawing, and storing of the serum samples did not interfere with obtaining valid results [24]. Further work to investigate the impact of sample storage may be an area for future research.

Another limitation of the current study was the use of plasma for RapidSTATUS™ FIV testing instead of fresh whole EDTA blood. According to the manufacturer, RapidSTATUS™ FIV can be tested with serum, plasma, or anticoagulated whole blood. In a previous study, testing of cats in the second study population using fresh EDTA blood with Witness^®^ and Anigen Rapid^®^ on day 42 (2 weeks following the second FIV vaccination in the primary course) produced the greatest number of false-positive results, suggesting this was the time of peak vaccine-induced antibody production [18]. To cursorily assess the possible variability in results with the use of plasma (as in the current study) and EDTA (as in the previous study), a small subset of samples from the second study population was re-tested with Witness^®^ using thawed plasma. There was agreement in 4/5 (80%) of tests (results not shown). Furthermore, in a previous study, results from Witness^®^ and Anigen Rapid^®^ tests with fresh whole EDTA blood and thawed plasma that had been stored at −80 °C were 99% in agreement [14]. For cats in the third study population, testing using fresh EDTA blood with Witness^®^ and Anigen Rapid^®^ did not produce any false-positive results [19], corresponding with RapidSTATUS™ FIV results using plasma in the current study. Further work to investigate possible differences between testing with fresh anticoagulated whole blood versus plasma and/or serum may be an area for future research.

Recently, an alarmingly high number of false-negative PoC test results were observed with Witness^®^ and SNAP^®^ Combo, mainly in samples originating from Switzerland. It was proposed that the introduction of new FIV isolates may have been the cause of the false-negative results in FIV-infected cats [25]. Therefore, it should be emphasized that results from this study apply to FIV testing in Australia, and results should not be extrapolated overseas without appropriate independent studies that report results in cats infected with locally circulating FIV strains.

## 5. Conclusion

The results of this study demonstrate that RapidSTATUS™ FIV can accurately determine the true FIV status of cats of known and unknown FIV vaccination history, including cats recently administered vaccines as part of their primary FIV vaccination course or an annual FIV vaccination booster. The high level of accuracy of RapidSTATUS™ FIV (98.8–100%), therefore, establishes this PoC kit as a DIVA test.

## Figures and Tables

**Table 1:** Results from RapidSTATUS™ FIV testing in pet cats in Australia with a range of FIV vaccination/infection statuses (*n* = 361). This comprised 57 FIV-uninfected cats annually vaccinated against FIV, 10 FIV-uninfected cats with lapsed FIV vaccination histories, 259 FIV-unvaccinated/FIV-uninfected cats, and 35 FIV-infected cats.

		**RapidSTATUS™ FIV Test Result**	
		+	−
FIV infection status	+	34	1
	−	0	326

**Table 2:** Results from RapidSTATUS™ FIV testing in FIV-vaccinated/FIV-uninfected cats in Australia. Cats were either administered a primary FIV vaccination course (3 vaccines on days 0, 28, and 56), or an annual booster FIV vaccination (1 vaccine on day 0). NA = not available.

Days after Vaccination	No. of False-Positive RapidSTATUS™ FIV Results	
	Primary FIV Vaccination	Annual FIV Vaccination
0	0/7	0/10
14	0/11	0/7
28	0/12	0/9
42	0/11	0/6
56	0/10	NA
70	1/12	NA
84	0/11	NA
98	0/2	NA
140	0/1	NA
238	0/5	NA
TOTAL	1/82	0/32

## Data Availability

All the data presented in this paper are available on request.
